# Rapid circulation of HIV-1 CRF85_BC in Southwest China: its geographic origins and molecular transmission networks analysis

**DOI:** 10.3389/fcimb.2025.1624996

**Published:** 2025-09-26

**Authors:** Yang Liu, Cuixian Yang, Wei Chang, Xiaoyang Fu, Mi Zhang, Li Gao, Li Liu, Xingqi Dong, Yue Feng, Xueshan Xia

**Affiliations:** ^1^ Faculty of Life Science and Technology, Kunming University of Science and Technology, Kunming, China; ^2^ Precision Medicine Research Institute, Kunming Medical University, Kunming, China; ^3^ Department of Clinical Laboratory, Yunnan Provincial Infectious Diseases Hospital, Kunming, China; ^4^ Yunnan Provincial Key Laboratory of Public Health and Biosafety, Kunming Medical University, Kunming, China

**Keywords:** HIV-1, CRF85_BC, origin, molecular transmission network, dynamic

## Abstract

**Background:**

Until December 2024, China had identified and named 66 new HIV-1 recombinant genotypes. Among them, CRF85-BC is showing a rapidly growing trend in popularity in southwestern China, especially in Sichuan and Yunnan. This genotype was first discovered and reported in Sichuan and is believed to have originated in Yunnan. However, there are relatively few reports on the comprehensive systematic transmission data in Yunnan. This study will further elucidate the accurate evolutionary origin time and epidemic transmission dynamics of CRF85-BC.

**Methods:**

We obtained 496 partial pol and 47 near full-length genomic sequences of HIV-1 CRF85_BC from 28,384 individuals with treatment failure in Yunnan, 2009-2023. Bayesian coalescent phylogeny analysis was performed to investigate the origin and timeline of CRF85_BC. A molecular transmission network was constructed using the genetic distance method to evaluate the transmission pattern. Spatial analysis was used to reveal the geographic patterns of phylogenetic clustering rates.

**Results:**

The number of CRF85_BC sample cases increased significantly between 2009 and 2023, and showed resistance to reverse transcriptase inhibitors (M184V/I and K103N/S). Bayesian phylogeny of nearly full-length sequences indicated that the emergence time in Yunnan was between January 1989 (95% confidence interval [CI]: 1984.9-1992.8) and February 1992 (95% CI: 1986.1-1996.6). Molecular networks resolved 87 transmission clusters, and the differences in transmission patterns were mainly manifested in the high aggregation rate (63.29%; 95% CI: 55.69%-70.89%) and cluster size (average size: 8.3) in Sichuan, which were higher than those in Yunnan (40.33%; 95% CI: 36.19%-44.47%; average size: 2.5). And all of them were heterosexual people, with a predominance of 77.81% (256/329). Spatiotemporal analysis revealed that Yunnan significantly transmitted to Sichuan, with Zhaotong and Yibin serving as key transmission hubs in both provinces.

**Conclusions:**

The CRF85_BC genotype from Yunnan has a growing transmission network, with the potential for further expansion.

## 1 Introduction

The HIV-1 virus is highly diverse and complex due to frequent mutations, recombination, and the emergence of drug-resistant strains (Taylor et al., 2008). In China, the major prevalent genotypes and recombinant types of HIV-1 are B, C, CRF01_AE, CRF07_BC, and CRF08_BC (Li et al., 2018; Vrancken et al., 2020). These genotypes and recombinant phenotypes can recombine with each other, resulting in the emergence of new recombinant strains. As of December 2024, 66 new HIV-1 recombinant genotypes have been identified and named in China. Among them, CRF85_BC has shown a rapidly increasing epidemic trend in southwest China, particularly in Sichuan and Yunnan (Su et al., 2020). Therefore, it is crucial to monitor the prevalence and transmission of HIV-1 CRF85_BC.

The CRF85_BC strain was initially identified in 2016 among heterosexual populations in Yibin City, Sichuan Province, China. Subsequently, reports emerged in other regions, including Yunnan, Chongqing, Anhui, Henan, and Zhejiang. In addition to Sichuan and Yunnan Provinces, a total of 20 CRF85_BC cases were identified among newly diagnosed infections in Zhejiang (2016), Fujian (2020), and Ningxia (2023), with the affected populations commonly characterized by older age and heterosexual transmission (Xie et al., 2023; Zhang et al., 2017; Li et al., 2025). In Sichuan, for instance, the prevalence of this strain increased from 3.39% in 2014 to 5.17% in 2019 (Su et al., 2020; Cao et al., 2023). Similarly, in Yunnan, the prevalence increased from 1.1% in 2015 to 6.5% in 2021. This particular strain seems more common among older heterosexual individuals over 50 and has established a large, tightly connected molecular transmission network, mainly marked by strong intercity spread (Zhou et al., 2021).

The HIV-1 strain CRF85_BC is currently being disseminated from Yibin City, Sichuan Province, to other regions within Sichuan and neighboring Yunnan Province (Zhaotong). A recent study posits that CRF85_BC may have originated in Yunnan, a southwestern Chinese province that shares a border with Myanmar, Laos, and Vietnam. The initial case of HIV-1 infection in mainland China was identified in the Ruili district of Yunnan Province in 1989 (Xiao et al., 2007). This region, particularly the border area between China and Myanmar, has been identified as a significant locus for developing novel recombinant HIV-1 strains (Wang et al., 2015; Chen et al., 2018). In Yunnan Province, 25 novel recombinant HIV-1 strains have been identified and designated for the first time.

Although some studies have documented the epidemiologic characteristics of CRF85_BC in Yunnan, these studies have limitations such as small sample sizes and the exclusive use of cross-sectional studies. Therefore, a more comprehensive molecular epidemiological investigation of CRF85_BC in Yunnan is needed. This will facilitate a systematic investigation of the molecular evolution of CRF85_BC and the characteristics of the molecular transmission network. Such research is crucial for the development of effective diagnostic and treatment strategies for CRF85_BC. In this study, we conducted a retrospective investigation of the prevalence of CRF85_BC among 28,384 HIV-positive patients receiving highly active antiretroviral therapy (HAART) in Yunnan between 2009 and 2023. A systematic analysis of the molecular evolution and molecular transmission network of CRF85_BC was performed by combining all available CRF85_BC sequences in the HIV database.

## 2 Methods

### 2.1 Study participants

A total of 28,384 Plasma samples and demographic data were collected from HIV-1 patients in Yunnan Province who had experienced treatment failure with HAART and exhibited a viral load of ≥1000 copies/mL between 2009 and 2023. All HIV-1 tests were informed and voluntary. The corresponding demographic characteristics include age, gender, transmission method, RNA viral load, etc. Plasma was separated from whole blood samples using EDTA tri-potassium salt and stored at −80 °C for HIV RNA extraction.

### 2.2 HIV-1 RNA extraction, partial pol gene amplification, and sequencing

HIV-1 RNA was isolated from 200-µl plasma samples using the MiniBEST Viral RNA/DNA Extraction Kit according to the procedure described in the manual. Then, the partial pol gene (HXB2: 2147-3462) fragments were amplified by nested polymerase chain reaction (PCR), and the PCR primers and conditions were reported in the previous study (Feng et al., 2018; Zhang et al., 2019). Amplified PCR products were detected by electrophoresis on a 1.0% agarose gel under ultraviolet illumination and purified using a DNA purification kit. The products were sequenced by TSINGKE Biological Technology Co. using an ABI 3730XL automated DNA sequencer.

### 2.3 HIV-1 genotyping and drug resistance analysis

The sequencing data were aligned against the HIV-1 sequence database using NCBI BLAST search and manually edited with Bioedit 7.2.1 regarding HXB2 to ensure accurate codon alignment. Then, the gag-pol gene sequences were submitted to an HIV-1 online Quality Control software (https://www.hiv.lanl.gov/content/sequence/QC/index.html) to confirm the sequence quality. Phylogenetic trees were constructed based on the obtained datasets with the maximum-likelihood method using MEGA v.6.0.6 and the general time reversible + gamma distribution + invariant sites (GTR+G+I) model (Tamura et al., 2013). Bootstrap values were calculated based on 1000 replications of the alignment. The reference sequences relevant to HIV-1 epidemics in Asia were downloaded from the Los Alamos National Laboratory HIV sequence database. All of the assembled partial pol genes were submitted to the Stanford University HIV Drug Resistance Database online sequence analysis tool (https://hivdb.stanford.edu/hivdb/by-sequences/) to confirm the genotyping and to identify the drug resistance mutations.

### 2.4 HIV-1 near full-length genome amplification, sequencing, and sequence analysis

The near full-length HIV-1 genome was amplified separately using reverse transcription (RT)-nested polymerase chain reaction according to the method described in previous reports (Grossmann et al., 2015; Zhang et al., 2019). The generated products were analyzed by agarose gel electrophoresis, and the positive PCR samples were purified using the PCR Product Gel Extraction Kit (Tiangen). Then, Sanger’s method of gene sequencing was performed by Invitrogen Co. (Guangzhou, China). Recombination breakpoints were determined using SimPlot 3.5.1 software to perform bootscanning and informative-site analyses. Based on the information generated from Simplot, the structure of the new HIV-1 recombinant forms (B/C) was elucidated using the Recombinant HIV-1 Drawing Tool.

### 2.5 Bayesian coalescent phylogeny construction

In this study, we employed a Bayesian phylogenetic approach to estimate the time to the most recent common ancestor (TMRCA) and evolutionary rates for a dataset. A GTR+G+I nucleotide substitution model and an uncorrelated relaxed molecular clock (UCLN) model were utilized. The UCLN model allows for the evolutionary rate of each branch in the tree to be distinct and independent of those of neighboring branches. Moreover, a Bayesian skyline coalescent tree prior was utilized to ascertain the historical population dynamics of rapidly evolving pathogens. This analysis was conducted using BEASTv1.10.4, a cross-platform software that employs the Markov chain Monte Carlo (MCMC) framework for Bayesian analysis of genetic sequence data, to estimate time-sampled phylogenies (Drummond et al., 2012; Suchard et al., 2018). The molecular clock rate was set with a continuous-time Markov chain (CTMC) reference prior, which was employed as a null hypothesis (Faria et al., 2014). To accelerate the Bayesian analysis, likelihood evaluation was parallelized using BEAGLE v3.1.2. The molecular clock was calibrated using a tip calibration approach. The MCMC chain was executed for 100 million states, with a sampling frequency of every 10,000 states, to ensure sufficient mixing of all model parameters, including trees. The convergence of the MCMC chain was evaluated using the Tracer v1.7.1 software (Rambaut et al., 2018). It was determined that all parameters had converged once their effective sample sizes reached a value of at least 200. The representative sequences annotated by the provinces were selected, and the Bayesian stochastic search variable selection (BSSVS) procedure was used to determine the propagation relationship of CRF85_BC among provinces (Lemey et al., 2009). SpreaD3 v0.9.6 software was used to calculate Bayesian factors (Bielejec et al., 2016), among which the results of Bayesian factors ≥ 3 are further analyzed, 3<BF ≤ 10 as substantial evidence, 10<BF ≤ 30 as strong evidence, 30<BF ≤ 100 as very strong evidence, and BF>100 as decisive evidence (Luo et al., 2024). The maximum clade credibility (MCC) summary tree was generated using TreeAnnotator v1.10.4 from the BEASTv1.10.4 software package, with the initial 10% of trees discarded as burn-in. The resulting MCC summary tree was then rendered in FigTree software for subsequent analysis.

### 2.6 Pairwise distance and transmission network analysis

The TN93 model was utilized to calculate genetic distances between all CRF85_BC sequences (Tamura and Nei, 1993). To construct the molecular network, a threshold was established that would result in the greatest number of clusters while simultaneously minimizing the genetic distance (Wertheim et al., 2018; Li et al., 2021; Peng et al., 2025; Su et al., 2025). Subsequently, only those sequences that fell within the identified clusters were included in the subsequent analysis. Subsequently, the network was rendered in Cytoscape v3.9.1 software for further analysis (Fan et al., 2021).

### 2.7 Spatial analysis

Each sequence in the molecular network is projected onto a map of its sample source location. The population size of each city included in the network will be represented by the radius of the circle, based on a matrix of city-to-city connectivity strength and flow maps. The associated sample source locations will be connected using flow maps, with the line strength scaled according to the number of connections. The varying widths of the lines will serve to indicate the number of connections between the two cities in question.

### 2.8 Statistical analysis

Chi-square and Bonferroni correction tests were used to compare the distribution of population information and transmission networks among groups, and a p-value less than 0.05 was considered statistically significant. SPSS v.20.0 software was used for statistical analysis.

### 2.9 GenBank accession numbers

The nucleotide sequences reported in this study have been submitted to GenBank with accession numbers PV184678 - PV185173.

### 2.10 Ethics statement

This study adhered to the tenets of the Declaration of Helsinki and was approved by the Yunnan Provincial Hospital of Infectious Diseases Ethics Committee, AIDS Care Center (Approval No. YNACCA [2015]-12). All participants provided written informed consent for sample collection and subsequent analysis.

## 3 Results

### 3.1 The rapid increase in CRF85_BC infections

**Figure 1:**
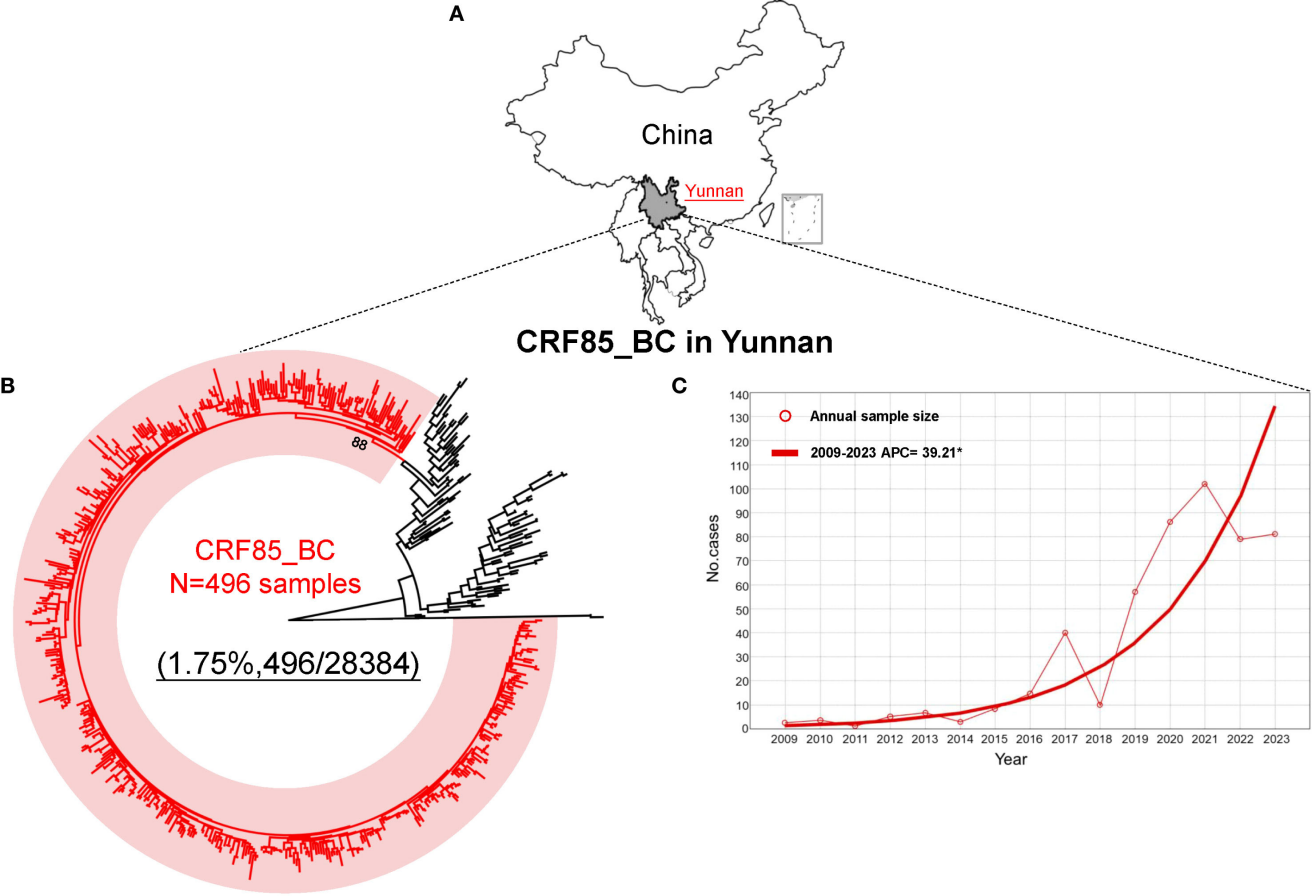
The source of CRF85_BC samples and the distribution trend of annual numbers. **(A)** The sequences provided in this study are all from Yunnan Province, China. **(B)** A total of 496 CRF85_BC sequences were identified based on phylogenetic tree analysis, with the red line representing CRF85_BC sequences and the black line representing other reference sequences. **(C)** Joinpoint regression analysis was performed on the sequences collected from 2009 to 2023, with the circles on the axis representing the number of samples each year, and the red solid line representing the APC fitted curve.

Phylogenetic analysis revealed that a total of 496 HIV-1 CRF85_BC pol sequences were obtained from individuals with virological failure of antiretroviral therapy (ART) in Yunnan between the years 2009 and 2023 (**Figures 1A, B**), and the joinpoint regression was employed to analyze the trend in the annual number of CRF85_BC cases. The result showed a significant increase in the number of individuals infected with HIV-1 CRF85_BC in Yunnan, with an average annual percent change of 39.21 (95% CI: 26.18–53.34) (**Figure 1C**), and the detailed information of 496 sequences was collected (**Supplementary Table S1**).

### 3.2 Drug resistance characteristics of CRF85_BC in Yunnan, China

**Figure 2:**
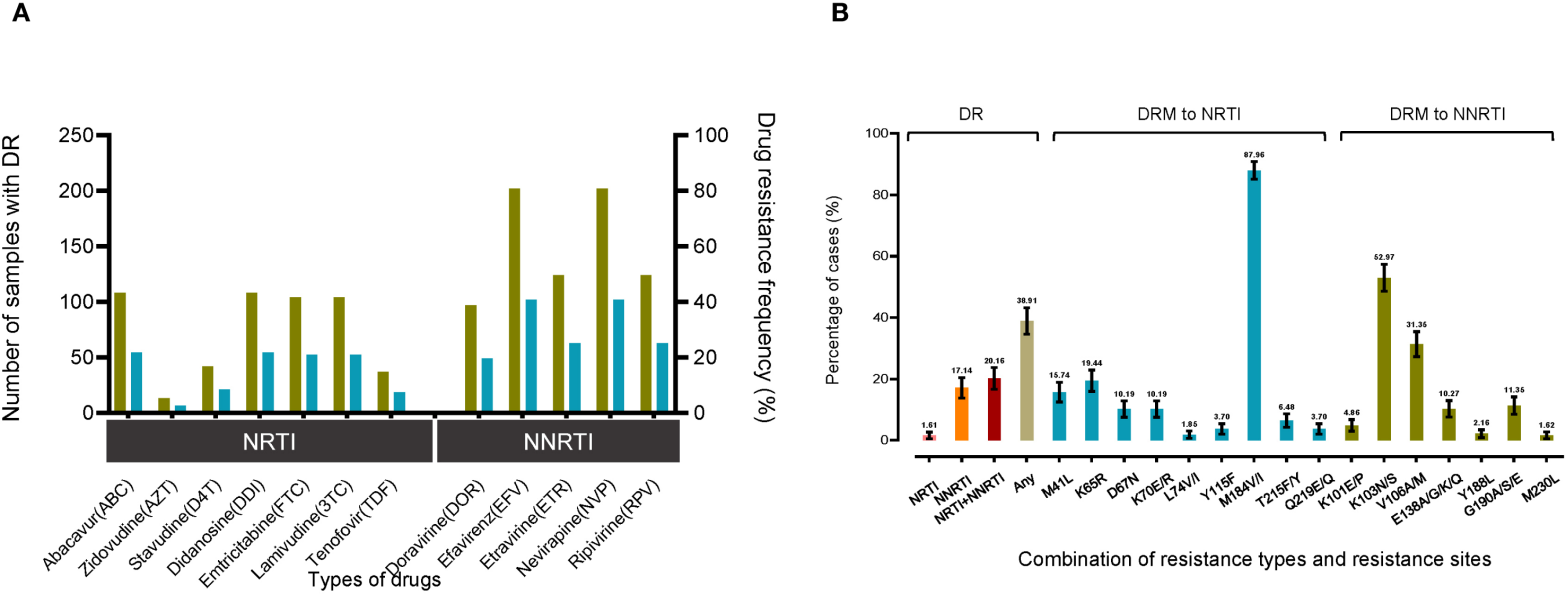
Drug resistance analysis of CRF85_BC sequences. The drug resistance analysis summarizes the drug types (nucleoside and non-nucleoside antiretroviral drugs) and the corresponding resistance sites. **(A)** The number and percentage of resistant samples in a total of 496 samples were counted. **(B)** The percentage distribution and summary were performed according to resistance types and Surveillance Mutations sites. "NRTI" represents the percentage of sequences containing only NRTIs, "NRTI+NNRTI" represents a drug resistance combination containing both NRTI and NNRTI and "any" refers to the percentage of sequences containing any type of drug.

The study examined the prevalence of drug resistance (DR) among different antiretroviral drug classes, including protease inhibitors (PIs), nucleoside reverse transcriptase inhibitors (NRTIs), and non-nucleoside reverse transcriptase inhibitors (NNRTIs). Among treatment failures, DR was identified in 38.91% of cases. Specifically, drug resistance was detected for the NRTIs Lamivudine(3TC) (20.97% [104/496]) and Abacavir (ABC) (21.77% [108/496]) and the NNRTIs Efavirenz (EFV) (40.73% [202/496]) and Nevirapine (RPV) (25.00% [124/496]). The prevalence of Zidovudine (AZT) in DR cases was low at 2.62% (13/496), despite its frequent use in ART regimens. Notably, no resistance to PIs was observed (**Figure 2A**).

The study further revealed that, among the categories of drug resistance combinations, the co-occurrence of NRTI and NNRTI mutations represented the largest proportion at 20.16% (95% CI: 16.63%-23.69%), followed by NNRTI mutations alone at 17.14% (95% CI: 13.82%-20.46%), and NRTI mutations alone at 1.61% (95% CI: 0.50%-2.72%). The prevalence of drug resistance mutations (DRMs) to NRTIs and NNRTIs was also examined for the period 2009-2023. The most common major NRTI mutation was M184V/I, observed in 87.96% (95/108) of cases, followed by K65R at 19.44% (21/108) and M41L at 15.74% (17/108). A total of 185 patients had a major DRM to NNRTIs, with K103N/S being the most common mutation observed in 52.97% (98/185) cases. Subsequently, V106A/M was identified in 31.35% (58/185) of patients, while G190A/S/E was observed in 11.35% (21/185) of cases (**Figure 2B**).

### 3.3 Estimation of origin time and distribution of CRF85_BC

**Figure 3:**
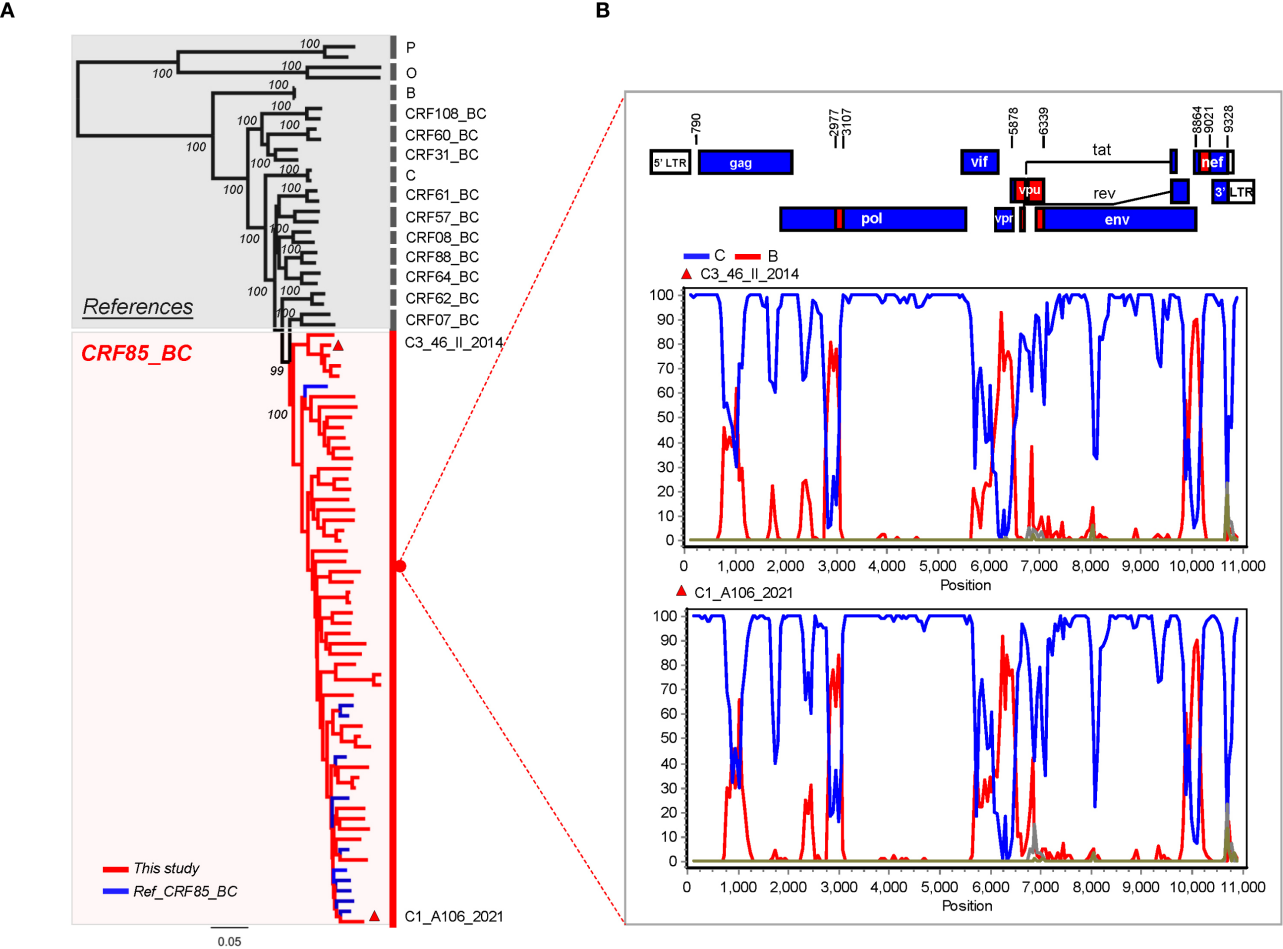
Recombination Pattern Analysis of the Near-Full-Length CRF85_BC Sequences. **(A)** In the phylogenetic tree, blue represents reference sequences from the database, while red represents the Yunnan sequences added in this study. **(B)** Two near-full-length sequences were randomly selected from panel A for CRF85_BC bootscanning validation. The window was moved in 500-nt increments, and the vertical axis represents the bootstrap values supporting clustering with subtype reference sequences. Subtype O was used as the outgroup.

To confirm the recombination pattern of CRF85_BC and elucidate the geographic origin of endemic CRF85_BC, near-full-length amplification (HXB2:638-9600) was initially performed on 50 randomly selected samples, resulting in 47 sequences. After combining these with 11 sequences from the database, a dataset of 58 sequences was formed. The phylogenetic tree and bootscanning results showed that the recombination pattern was consistent with the previously accepted findings (**Figures 3A, B**).

**Figure 4:**
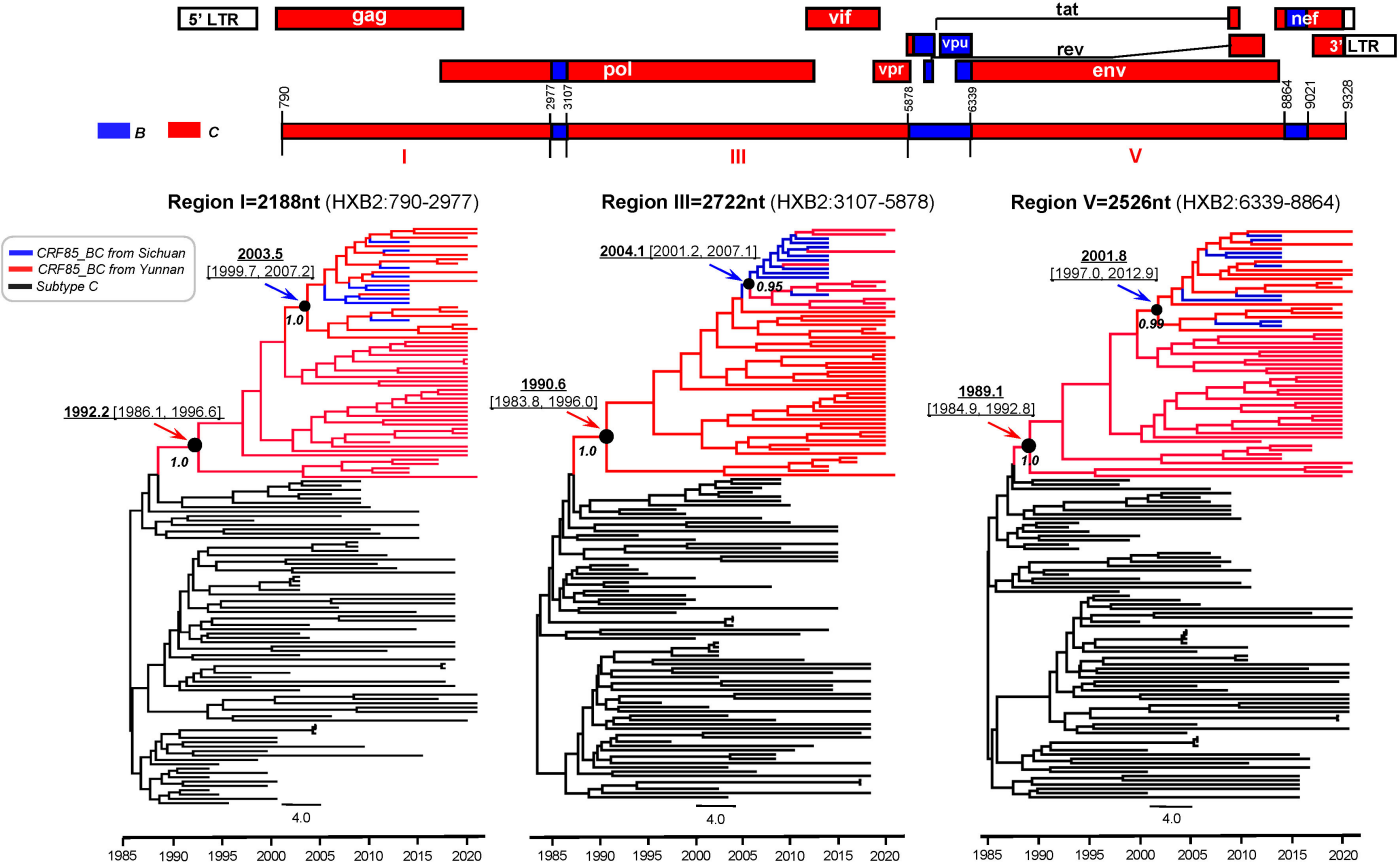
Bayesian maximum clade-credibility tree for HIV-1 CRF85_BC C segments in Yunnan and Sichuan. The MCC tree was constructed based on the three large C segments of the near-full-length CRF85_BC sequences, including sequences from Sichuan (blue) in the database and those from Yunnan (red) provided in this study. The TMRCA is indicated with an arrow.

**Figure 5:**
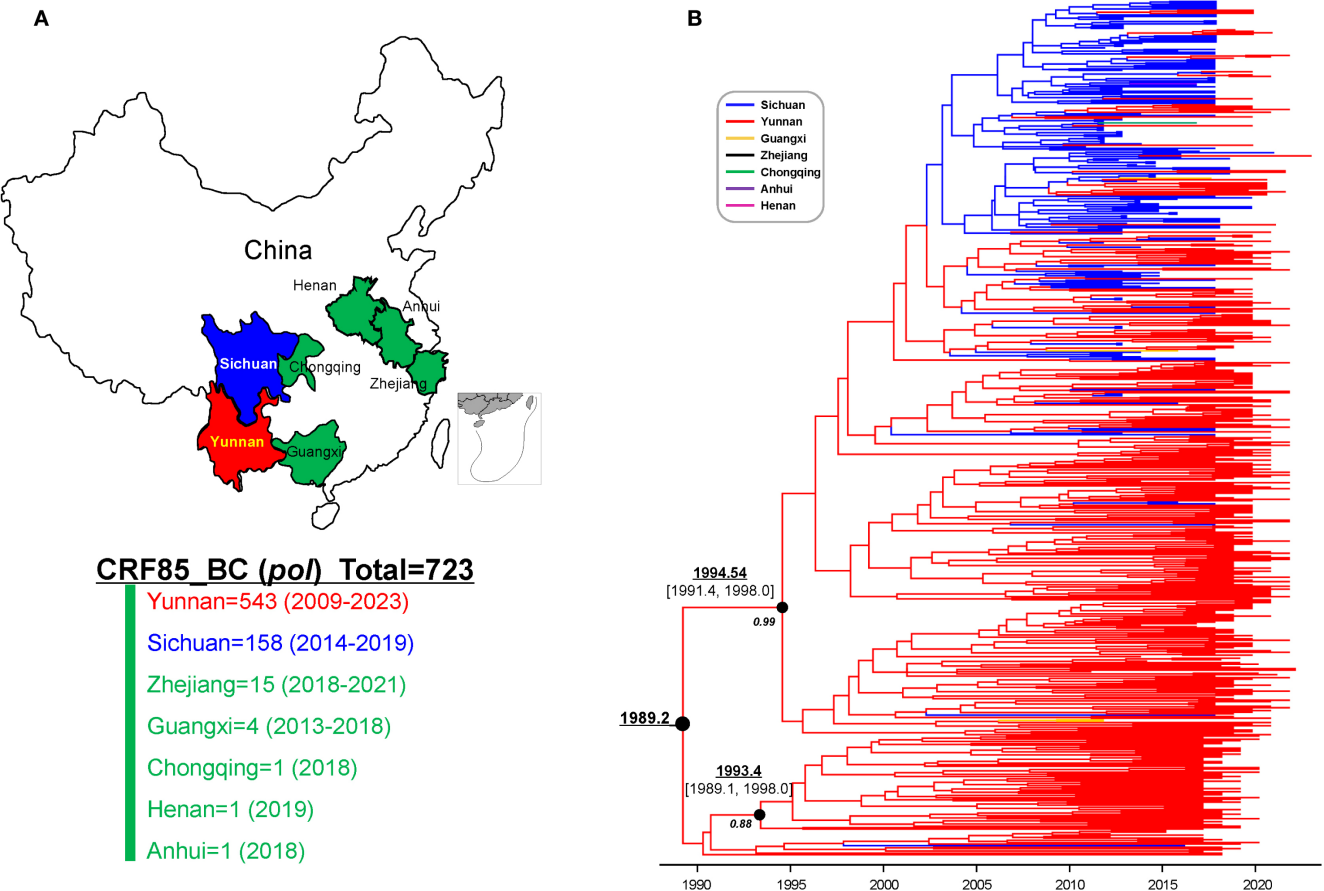
The spatiotemporal distribution of HIV-1 CRF85_BC sequences. **(A)** Geographical and temporal distribution of CRF85_BC pol region sequence collections from 2009 to 2023. **(B)** The maximum clade-credibility tree constructed in BEAST v1.10.4 using the GTR+I+G substitution model, log-normal relaxed molecular clock model, and non-parametric Bayesian SkyGrid model. The branch colors represent the sampling locations.

Bayesian molecular clock analysis of the near full-length sequence confirmed that the origin time was between 1989.1 (95% CI: 1984.9-1992.8) and 1992.2 (95% CI: 1986.1-1996.6) (**Figure 4**). Subsequently, all previously reported CRF85_BC pol region sequences (HXB2:2253-3312) and the newly selected sequences from Yunnan were compiled, forming a dataset of 723 sequences from 7 provinces and cities in China (**Figure 5A**). Phylogenetic analysis of pol sequences estimated CRF85_BC’s earliest transmission to 1989.2, consistent with its near full-length genomic origin timeframe. In addition, the MCC tree delineated two major lineages: the root lineage (posterior probability=0.88), composed of early Yunnan sequences, diverged in 1993.4[95% highest posterior density [HPD]: 1989.1–1998.0], while the crown lineage (posterior probability=0.99), containing sequences from Yunnan and other Chinese provinces, diverged in 1994.54[95% HPD:1991.4-1998.0] (**Figure 5B**). BSSVS analysis further confirmed the high connectivity between Sichuan and Yunnan sequences, supporting the transmission event from Yunnan to Sichuan (Bayes Factor=23,735, posterior probability=1.00). Additionally, CRF85_BC strains originating in Yunnan have spread to Zhejiang, Guangxi, and Henan, while those from Sichuan disseminated further to Chongqing, Guangxi, and Anhui (**Supplementary Figure S1**; **Supplementary Table S2**).

### 3.4 The characteristics and dynamics of CRF85_BC molecular transmission networks

**Figure 6:**
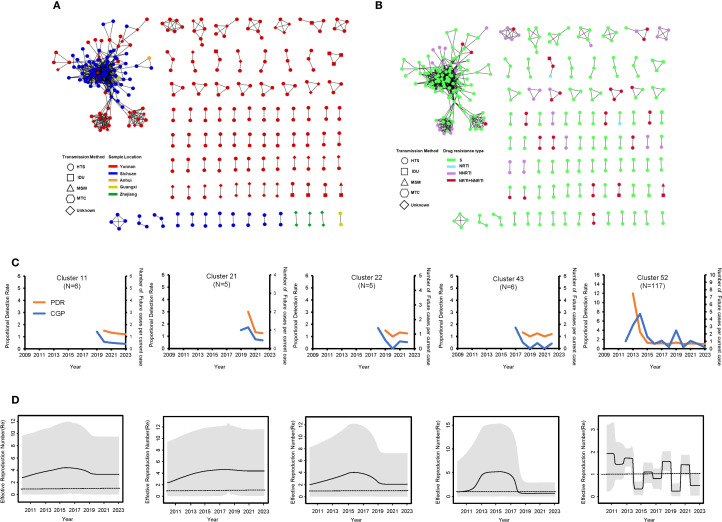
Molecular transmission network and dynamics analysis of CRF85_BC. **(A)** Transmission network by region and transmission route. **(B)** Transmission network by drug resistance type and transmission route. **(C)** PDR (orange line) and CGP trend (blue line) for medium and large transmission clusters during the period 2009–2023. **(D)** Re values of medium and large transmission clusters during the period 2009–2023. The Re value was inferred from the birth-death skyline plot. The thick solid line represents the estimated mean, and the 95% HPD are shown as gray areas.

A total of 87 clusters were identified in the molecular network with a genetic distance threshold of 0.007 substitutions/site, involving 329 sequences. The cluster sizes ranged from 2 to 117 (**Supplementary Figure S2**). Among the 87 clusters, 82 clusters (94.25%) contained 2 to 4 sequences, while 5 clusters (5.75%) contained 5 or more sequences. Further analysis based on the network population structure and scale structure revealed significant differences in characteristics such as age composition (χ² = 46.199, p < 0.05), gender (χ² = 43.348, p < 0.05), and transmission route (χ² = 9.306, p < 0.05). Specifically, in terms of geographical distribution, transmission clusters formed by Yunnan sequences were smaller and more dispersed, while Sichuan sequences formed larger clusters, with both higher clustering rates and larger average cluster sizes (**Figure 6A**; **Table 1**). Additionally, 29 molecular transmission clusters carrying drug-resistant mutations were identified in the transmission network, exhibiting resistance to various classes of antiretroviral drugs (**Figure 6B**). This drug resistance also showed geographical differences; specifically, transmission clusters from Yunnan had a higher proportion of resistance compared to those from Sichuan (32.88% vs. 5.00%) (**Supplementary Table S3**).

**Table 1:** Demographic characteristics of 329 individuals in the network.

Classification	Population in cluster (N=329)	Cluster Size		χ2	P-value
		Small cluster (2–4 nodes)	Large cluster (≥5 nodes)		
	N (%)	N (%)190	N (%)139		
Age					
≤29	11 (3.34%)	9*^a^* (4.74%)	2*^a^* (1.44%)	46.199	<0.05*
30-49	95 (28.88%)	74*^a^* (38.95%)	21*^b^* (15.11%)		
≥50	36 (10.64%)	60*^a^* (31.58%)	32*^a^* (23.02%)		
Unknown	131 (39.82%)	47*^a^* (24.74%)	84*^b^* (60.43%)		
Gender					
Male	142 (43.16%)	100*^a^* (52.63%)	42*^b^* (30.22%)	43.348	<0.05*
Female	56 (17.02%)	43*^a^* (22.63%)	13*^b^* (9.35%)		
Unknown	131 (39.82%)	47*^a^* (24.74%)	84*^b^* (60.43%)		
Route of infection					
IDU	10 (3.04%)	10*^a^* (5.26%)	0*^a^* (0.00%)	9.306	<0.05*
HET	256 (77.81%)	140*^a^* (73.68%)	116*^b^* (83.45%)		
Other	2 (0.61%)	1*^a^* (0.53%)	1*^a^* (0.72%)		
Unknown	61 (18.54%)	39*^a^* (20.53%)	22*^a^* (15.83%)		
Drug Resistance					
S	252 (76.60%)	136*^a^* (71.58%)	116*^b^* (83.45%)	17.915	<0.05*
NRTI	2 (0.61%)	2*^a^* (1.05%)	0*^a^* (0.00%)		
NNRTI	39 (11.85%)	20*^a^* (10.53%)	19*^a^* (13.67%)		
NRTI+NNRTI	36 (10.94%)	32*^a^* (16.84%)	4*^b^* (2.88%)		
Virus Load					
<1000	9 (2.74%)	4*^a^* (2.11%)	5*^a^* (3.60%)	43.003	<0.05*
1000-5000	42 (12.77%)	33*^a^* (17.37%)	9*^b^* (6.47%)		
5000-10000	18 (5.47%)	13*^a^* (6.84%)	5*^a^* (3.60%)		
10000-50000	73 (22.19%)	52*^a^* (27.37%)	21*^b^* (15.11%)		
50000-100000	25 (7.60%)	16*^a^* (8.42%)	9*^a^* (6.47%)		
≥100000	39 (11.85%)	28*^a^* (14.74%)	11*^a^* (7.19%)		
Unknown	123 (37.39%)	44*^a^* (23.16%)	79*^b^* (56.83%)		
Collected Province					
Sichuan	100 (30.40%)	26*^a^* (13.68%)	74*^b^* (53.24%)	59.661	<0.05*
Yunnan	219 (66.57%)	156*^a^* (82.11%)	63*^b^* (45.32%)		
Other	10 (3.03%)	8*^a^* (4.21%)	2*^a^* (1.44%)		

Factors not listed in the table are defined as "other," and information that cannot be obtained is defined as "unknown." In significance difference statistics, "*" and consistent superscripts in the upper-right corner of groups indicate no statistical significance, while inconsistent superscripts indicate statistical significance. The Chi-square test was performed by Bonferroni method, and the Chi-square test was performed for the data between the condition groups. P < 0.05 represents a statistically significant difference. In the statistics between groups, *a* represents no statistical difference (P > 0.05), and *b* represents statistically significant difference (P < 0.05).

The analysis focused on five clusters with nodes ≥5 in the transmission network, showing that the formation of four of these clusters was primarily driven by the introduction of new sequences after 2017, and this growth trend was consistent with the overall network expansion (**Supplementary Figure S2**). Both proportion detection rate (PDR) and cluster growth predictor (CGP) exhibited a general downward or fluctuating downward trend. Although there were slight differences in transmission capacity among the groups, the PDR values for all groups remained above 1, indicating that the transmission potential is still ongoing. This trend is also reflected in the birth-death serial model; clusters 11, 21, and 43 experienced rapid growth before 2017, reaching peak Re values, after which they began to decline post-2017 (**Figures 6C, D**). However, the effective reproduction number (Re) value for clusters 11 and 22 remained above 1, while the Re value for cluster 43 dropped below 1 after 2018 and has remained below 1 ever since. Additionally, cluster 11 maintained a Re value consistently around 4 after peaking before 2017. In contrast, the dynamics of cluster 52 (a very large transmission cluster) were significantly different from those of the other clusters. This cluster is associated with the transmission network between Yunnan and Sichuan. Specifically, the CGP and Re of cluster 52 fluctuated between 2014 and 2023. More precisely, the Re exceeded 1 during several periods: before 2014, during 2015-2016, during 2018-2019, and between 2020-2022, with a peak value of 1.897 (95% HPD: 0.178, 3.188). However, during the 2019–2020 period, the Re dropped to its lowest value of 0.273 (95% HPD: 0.016, 0.585), and this low value seems to have persisted to the present.

### 3.5 Spatial analysis among cities

**Figure 7:**
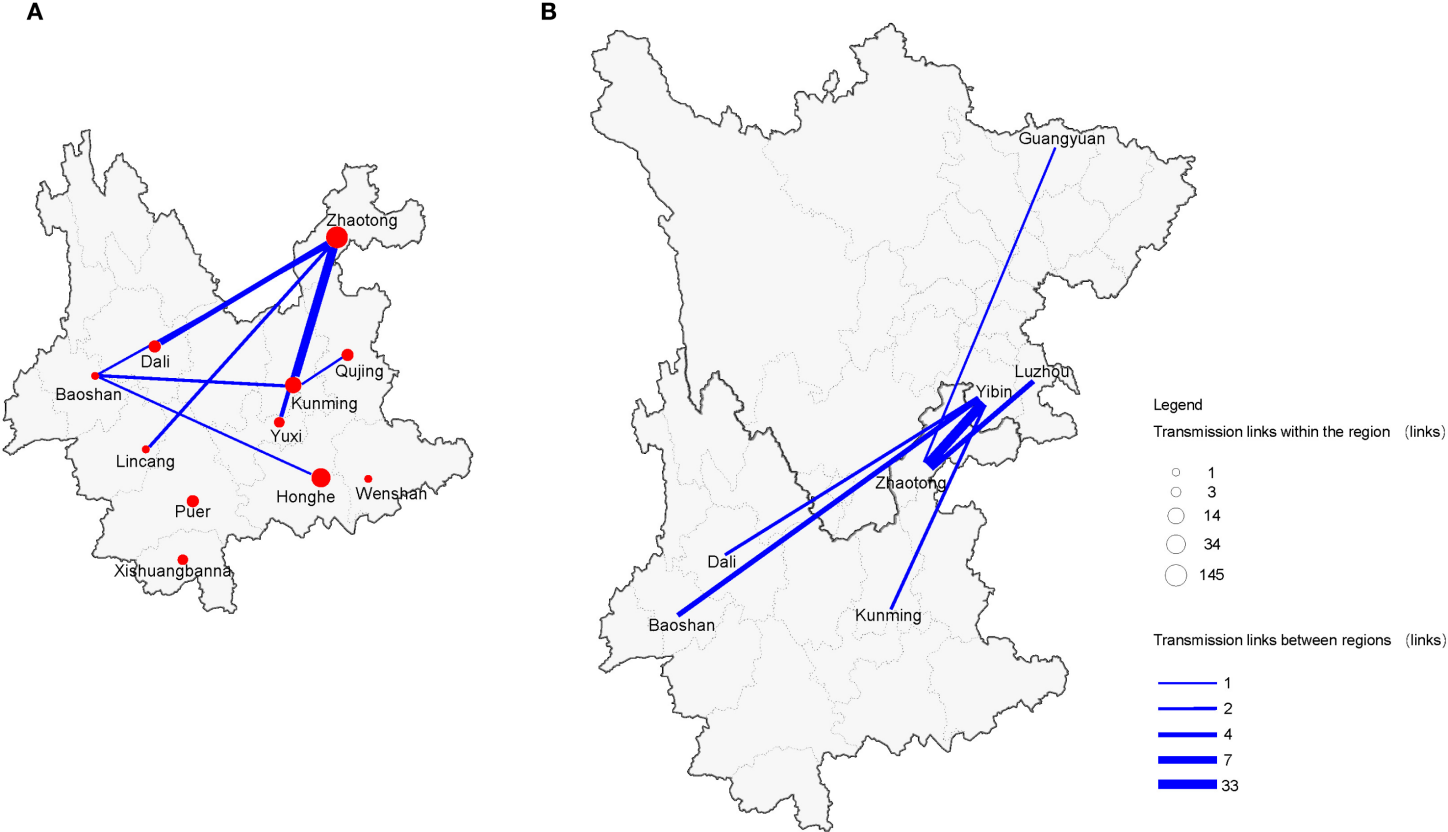
CRF85_BC spatial network transmission. **(A)** Intra and inter-city transmission within Yunnan Province. **(B)** Inter-provincial city transmission between Yunnan and Sichuan Provinces. Different sizes of circles represent the number of individuals in the network within the region; the line width indicates the number of transmission-related connections between regions.

In the spatial analysis, the transmission intensity of CRF85_BC within Yunnan Province exhibited significant differences between intra-city and inter-city transmission. We found that intracity transmission existed in 11 cities within Yunnan, with the strongest transmission chain observed in Zhaotong (145 links), followed by Honghe (34 links) and Kunming (14 links), while the intracity transmission strength in other cities was relatively weak. In terms of inter-city transmission, fewer cities were involved, with only 8 cities participating. Notably, Zhaotong and Kunming, as the two main regions for inter-city transmission, showed some similarity with intracity transmission. The strongest intercity transmission was between Zhaotong and Kunming (7 links), followed by Zhaotong and Dali (4 links), and Zhaotong with three other cities (**Figure 7A**).

Additionally, interprovincial transmission was also captured in this study. Zhaotong, Kunming, Dali, and Dehong, as major transmission cities within Yunnan, also participated in cross-provincial transmission with Yibin in Sichuan. The strongest interprovincial transmission occurred between Yibin and Zhaotong (33 links). As one of the important transmission cities for CRF85_BC in Yunnan, Zhaotong not only had a transmission link with Yibin but also showed relatively weaker transmission links with Luzhou and Guangyuan (**Figure 7B**).

## 4 Discussion

In recent years, Yunnan has emerged as a significant epicenter for the emergence of novel circulating recombinant forms (CRFs) of HIV, playing a critical role in the transmission dynamics of the virus (Ye et al., 2022). This study aims to comprehensively analyze the epidemiological evolution of HIV-1 CRF85_BC in China. Through extensive data collection and screening, our research has confirmed that the CRF85_BC strain originated in individuals in Yunnan Province, who became infected with HIV through heterosexual transmission, around 1989. The strain began to spread to Sichuan around 1994, and then spread and migrated to Zhejiang, Guangxi, and Henan, while Sichuan spread to Anhui and Chongqing, and Guangxi, similar to the earlier emergence of CRF07_BC and CRF08_BC in Yunnan following the reintegration of local sequences (Tee et al., 2008; Feng et al., 2016; Chen et al., 2018).

A comprehensive evaluation of the growth trends for CRF85_BC was conducted by examining epidemic clusters with dynamic parameters. The PDR and CGP were plotted alongside the Re for medium and large transmission clusters (Novitsky et al., 2015; Wertheim et al., 2018). Sequences were collected from a treatment-failure population in Yunnan, which is at higher risk of transmission and meets intervention criteria set by the China Centers for Disease Control and Prevention. The analysis identified three medium-sized clusters within these clusters. Notably, a general downward trend in PDR and CGP was observed, yet the Re values remained above 1, indicating ongoing potential for HIV transmission (Ragonnet-Cronin et al., 2018; Jovanović et al., 2019). Large transmission clusters exhibited notable declines in growth rates, particularly between 2014 and 2015, likely attributable to centralized treatment management. However, increasing drug resistance has diminished the effectiveness of population-wide treatment. A further decline was noted between 2019 and 2020, likely influenced by biases from the COVID-19 pandemic affecting population movement and data collection. While the growth rate of CRF85_BC in the Yunnan cluster has slowed, case numbers may still rise. The emergence of ultra-large transmission clusters from regions such as Yunnan, Sichuan, Anhui, and Guangxi introduces a degree of uncertainty regarding the future spread of CRF85_BC in China (**Supplementary Figure S2**).

The initial documentation of CRF85_BC in Sichuan has led to effective mapping of its associated outbreaks through molecular network analysis, allowing predictions of its evolution. Although this genotype has been less frequently reported in other regions of China, sequences from Yunnan show notable differences in network characteristics. Specifically, the overall cluster formation rate and average cluster size are smaller, and most sequences are more recent, especially those collected after 2017 (**Supplementary Figure S2**). Zhaotong was identified as a critical area for CRF85_BC, contributing significantly to both intra- and inter-urban transmission within Yunnan. This finding is consistent with previous studies that identified Zhaotong as a central location for epidemics associated with CRF01_AE and CRF08_BC, linking Yunnan with Guizhou and Sichuan (Cao et al., 2023). The risk of genetic recombination is heightened in Zhaotong, where CRF07_BC coexists predominantly with CRF85_BC, CRF101_01B, and CRF125_0107 (Zhou et al., 2022). Within Zhaotong, Shuifu, Yanjin, and Weixin counties, there is an abundance of evidence indicating that they are primary distribution areas for CRF85_BC and play an important role in interregional transmission dynamics with Yibin. This study integrates data from individuals experiencing HAART treatment failure in Yunnan, providing a more nuanced understanding of the CRF85_BC transmission network and its connections to Sichuan. Key sites of cross-provincial transmission include Yibin, Luzhou, and Guangyuan in Sichuan, and Zhaotong, Kunming, Dali, and Baoshan in Yunnan, with Yibin and Zhaotong identified as major sources of transmission. The results of this study provide valuable insights for the development of targeted detection and intervention strategies for CRF85_BC. The present study is not without its limitations. The research cohort did not include newly diagnosed cases or individuals who were ART-naïve during the same period, which limited the overall scope of the study and may have resulted in an underestimation of the true extent of transmission. In future, active collaboration with local CDC systems is intended in order to gain more comprehensive insights for developing targeted monitoring and intervention strategies for CRF85_BC.

In conclusion, this phylogenetic transmission analysis study confirmed that CRF85_BC originated in Yunnan and has been spreading at a relatively rapid pace in recent years. This study contributes valuable information for the future prevention and control of HIV-1.

## Data Availability

The datasets presented in this study can be found in online repositories. The names of the repository/repositories and accession number(s) can be found in the article/**Supplementary Material**.

## Supplementary material

Supplementary Figure 1 The spreading direction of CRF85_BC strain in China. The arrow represents the transmission direction. The width of the line indicates the size of Bayesian factor.

Supplementary Figure 2 Annual variation of molecular transmission clusters. The x-axis represents the generation time of clusters, and the y-axis arranges the numbers of each cluster. Different colors in each communication cluster represent sequences from different urban sources. The circle size represents the cumulative number of sequences in the cluster in the current year, the solid circle represents the cluster with continuous and active growth, and the hollow stroke cluster indicates that the cluster no longer has growth capacity.
