# Supplementary figures and images for: Rapid circulation of HIV-1 CRF85_BC in Southwest China: its geographic origins and molecular transmission networks analysis
Source: Front Cell Infect Microbiol. 2025 Sep 26;15:1624996. doi: 10.3389/fcimb.2025.1624996 (PMC12510952; doi:10.3389/fcimb.2025.1624996)

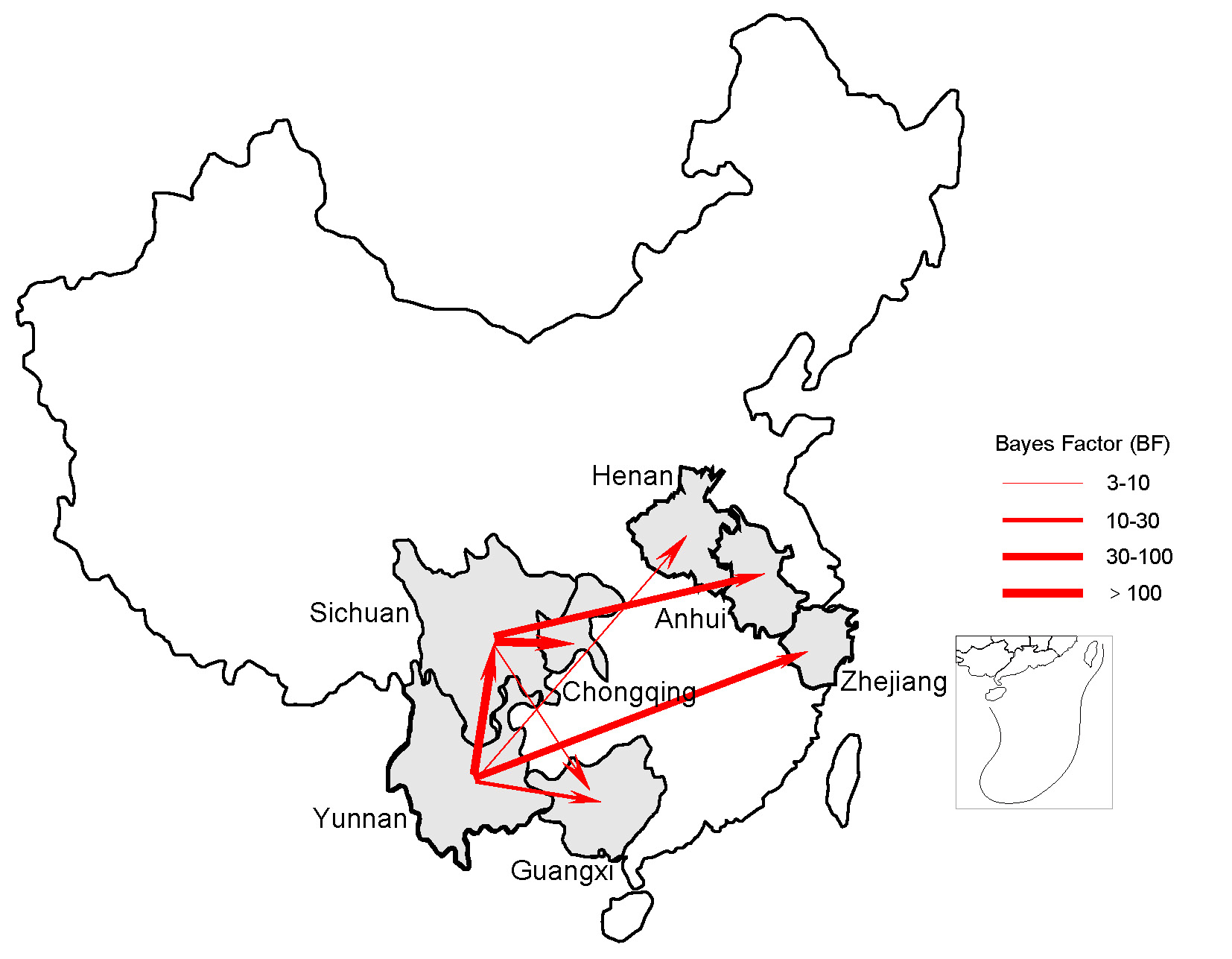

Supplement: Supplementary Figure 1 — The spreading direction of CRF85_BC strain in China. The arrow represents the transmission direction. The width of the line indicates the size of Bayesian factor. [file Image1.jpeg]

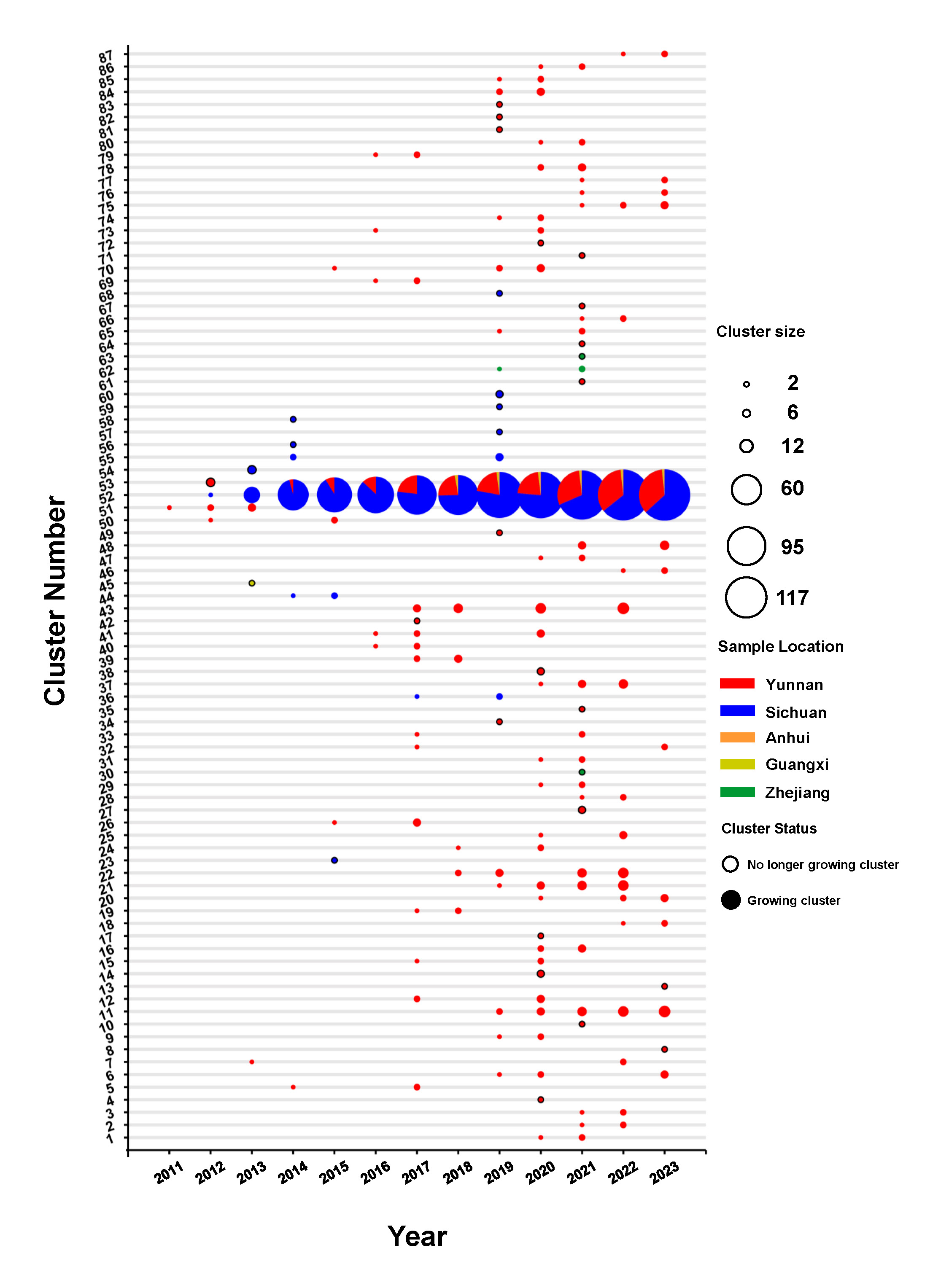

Supplement: Supplementary Figure 2 — Annual variation of molecular transmission clusters. The x-axis represents the generation time of clusters, and the y-axis arranges the numbers of each cluster. Different colors in each communication cluster represent sequences from different urban sources. The circle size represents the cumulative number of sequences in the cluster in the current year, the solid circle represents the cluster with continuous and active growth, and the hollow stroke cluster indicates that the cluster no longer has growth capacity. [file Image2.jpeg]
