# Supplementary material for: Rapid circulation of HIV-1 CRF85_BC in Southwest China: its geographic origins and molecular transmission networks analysis
Source: Front Cell Infect Microbiol. 2025 Sep 26;15:1624996. doi: 10.3389/fcimb.2025.1624996 (PMC12510952; doi:10.3389/fcimb.2025.1624996)
Supplement: Supplementary file 3 [file Table1.docx]

| **Characteristics** | **N** | ***p*-value** |
| --- | --- | --- |
| **Age** | | |
| <18 | 8（1.61%） | ＜0.05* |
| 18-60 | 280（56.45%） |  |
| >60 | 136（27.42%） |  |
| Other | 72（14.52%） |  |
| **Gender** | | |
| Male | 321（64.72%） | ＜0.05* |
| Female | 150（30.24%） |  |
| Unknown | 25（5.04%） |  |
| **Biochemical index of patients** | | |
| Virus Load，log10IU/mL | 5.18±2.85 | - |

**Table S1 The clinical demographic characteristics of 496 subjects**

**P* < 0.05 was considered statistically significant; groups were compared with the Chi-squared statistical test.
