# Supplementary material for: Rapid circulation of HIV-1 CRF85_BC in Southwest China: its geographic origins and molecular transmission networks analysis
Source: Front Cell Infect Microbiol. 2025 Sep 26;15:1624996. doi: 10.3389/fcimb.2025.1624996 (PMC12510952; doi:10.3389/fcimb.2025.1624996)
Supplement: Supplementary file 4 [file Table2.docx]

**Table S2. Results of BSSVS in different provinces of China**

| From | To | Bayes factor* | Posterior probability |
| --- | --- | --- | --- |
| Yunnan | Sichuan | 23735.1996 | 1.00 |
| Sichuan | Chongqing | 269.1812797 | 0.98 |
| Sichuan | Anhui | 59.23708452 | 0.92 |
| Yunnan | Zhejiang | 36.81796528 | 0.88 |
| Yunnan | Guangxi | 18.48916399 | 0.78 |
| Sichuan | Guangxi | 8.412140228 | 0.62 |
| Yunnan | Henan | 6.724154129 | 0.56 |

*The transmission relationships with a Bayes factor of >3 were selected. BSSVS, Bayesian stochastic search variable selection
