# Supplementary material for: Rapid circulation of HIV-1 CRF85_BC in Southwest China: its geographic origins and molecular transmission networks analysis
Source: Front Cell Infect Microbiol. 2025 Sep 26;15:1624996. doi: 10.3389/fcimb.2025.1624996 (PMC12510952; doi:10.3389/fcimb.2025.1624996)
Supplement: Supplementary file 5 [file Table3.docx]

**Table S3. Drug resistance characteristics of transmission network**

| **Province**  **(Total number of sequences entering network)** | **Number of drug resistance in the network**  **(Drug resistance ratio)** | **Network resistance cases** | | | **χ2** | **P-value** |
| --- | --- | --- | --- | --- | --- | --- |
|  |  | **NRTI** | **NNRTI** | **NRTI + NNRTI** |  |  |
| Yunnan（219） | 72（32.88%） | 2 | 36 | 34 | 0.811 | - |
| Sichuan（100） | 5（5.00%） | 0 | 3 | 2 |  |  |
| Other（10） | 0（0） | 0 | 0 | 0 |  |  |

Groups were compared with the Bonferroni correction test.
